# Reply to ‘Malignant pericardial effusion: sclerotherapy or local chemotherapy?’

**DOI:** 10.1038/sj.bjc.6605223

**Published:** 2009-08-11

**Authors:** H Kunitoh, T Tamura, N Saijo

**Affiliations:** 1 Department of Respiratory Medicine, Mitsui Memorial Hospital, 1 Kandaizumi-cho, Chiyoda-ku, Tokyo, Japan; 2 Department of Medical Oncology, National Cancer Center Hospital, Tokyo, Japan; 3 Department of Medical Oncology, Kinki University School of Medicine, Osakasayama, Japan

**Sir,**

We agree with Lestuzzi *et al* (2009) that our results on treatment using pericardial sclerosis with bleomycin in patients with lung cancer-associated malignant pericardial effusion (MPE), which was recently published in the *British Journal of Cancer* (Kunitoh *et al*, 2009), are unsatisfactory and leave much room for improvement. Although the acute toxicity/morbidity of the procedure was low, there were two cases with possible late pericardial constriction. Moreover, although our data did show a definite tendency for better control of MPE with pericardial sclerosis as compared with drainage alone, the primary end point of the phase III trial, survival without recurrent MPE at 2 months, was not statistically met. Therefore, the therapeutic advantage seems modest.

Having said that, we would like to rebut the points raised by Dr Lestuzzi *et al* on our paper.

First, they claim that the study subjects might not have had MPE, as there were some patients with cytology-negative effusion. It is well known that cytology is not always positive in MPE; in fact, according to Press and Livingston (1987), effusion cytology was positive in 151 (79%) of the 190 cases with pericardiocentesis-diagnosed MPE. That positivity rate was similar to that of our report, in which 57 (75%) of the cases examined for effusion cytology had positive results. With moderate or massive pericardial effusion clinically indicated for drainage in patients with confirmed lung cancer, the clinical diagnosis of MPE should be justified unless other findings suggest otherwise.

We do not rule out the possibility that there were a few cases with non-malignant effusion, and we do recognise that even among MPE patients cytology-positive cases should have larger tumour burden and worse prognosis (Gornik *et al*, 2005). Therefore, we performed a subset analysis (shown in Figures 2 and 3 of our paper), which showed the superiority of pericardial sclerosis in both cytology-positive and -negative cases. It would be clinically unjustified to wait for the results of repeated cytology in the management of clinically diagnosed MPE. We put priority on the clinical management of these patients with dismal disease, and not on the precision of biology or terminology.

On the second point, we agree with Dr Lestuzzi *et al* that previous reports, including that of ours, do not differentiate intrapericardial therapies according to the instilled agents, which range from purely ‘sclerotic’ agents, such as tetracycline, to almost purely chemotherapeutic agents, such as platinum. However, we understand that many of the previous trials are those on pericardial sclerosis and not on local chemotherapy; thiopeta, vinblastine and bleomycin have minimal activity against advanced lung cancers. Pericardial ‘local’ chemotherapy should be considered to be a relatively new strategy.

Finally, we must comment on Dr Lestuzzi *et al*'s claim regarding the ‘local chemotherapy’ with intrapericardial platinum.

First, we do not believe that it would be realistic to expect or try to ‘cure’ MPE in patients with far-advanced lung cancer. The aim of the management should be good and lasting palliation.

Of course, as we stated above, our results leave much room to be improved. Overall, the therapeutic advantage seems modest. Although massive bleeding upon re-attempted drainage occurred in two patients who did not take intrapericardial bleomycin, we did observe two cases with late pericardial constriction on taking bleomycin, which could be of more concern as improvement of lung cancer treatment yields more long-term survivors. There is certainly the possibility that intrapericardial platinum, as Dr Lestuzzi *et al* claim, may bring additional benefits to the patients.

However, their data, as well as those of others in the medical literature, are only from phase II trials or case series. In many of the trials, they treated various tumours with various agents, without any controlled data whatsoever. Their results could be useful as hypothesis-generating findings, but establishment of a standard therapy requires well-designed controlled trials.

We urge Dr Lestuzzi *et al* to conduct a phase III trial based on their rationale for intrapericardial platinum. In fact, that exactly is what we proposed as the next trial in the Discussion section. If they remain unconvinced with our data, they could put drainage alone for control. Phase III trials are much more difficult to conduct, but that is the only way in which they can demonstrate the superiority of their strategy.

In conclusion, we appreciate Dr Lestuzzi *et al* for their interest in our paper and for their effort towards improving the management of patients with MPE. However, phase II data cannot substitute for a randomised controlled trial to make medical evidence. They need to conduct a better phase III trial than ours to validate their claim.
